# Quantitative ctDNA Profiling of *RAS* Mutations as a Prognostic Biomarker in Metastatic Colorectal Cancer

**DOI:** 10.3390/ijms27010008

**Published:** 2025-12-19

**Authors:** Benoist Chibaudel, Elisabeth Carola, Hamid Mekranter, Perrine Goyer, Arnaud Saget, Olivier Oberlin, Hélène Marijon, Hubert Richa, Ida Iurisci, Honorine Gervais, Nathalie Perez-Staub, Linda Dainese, Pascal Pujol, Alain Toledano, Jean-Baptiste Bachet, Aimery de Gramont

**Affiliations:** 1 Department of Medical Oncology, Hôpital Franco-Britannique—Fondation Cognacq-Jay, Cancérologie Paris Ouest, 92300 Levallois-Perret, France; helene.marijon@cognacq-jay.fr (H.M.); ida.iurisci@cognacq-jay.fr (I.I.); honorine.gervais@cognacq-jay.fr (H.G.); nathalie.perez-staub@cognacq-jay.fr (N.P.-S.); aimerydegramont@gmail.com (A.d.G.); 2 Department of Medical Oncology, Groupe Hospitalier du Sud de l’Oise, 60100 Creil, France; elisabeth.carola@ghpso.fr; 3 Department of Medical Oncology, Centre Hospitalier François Quesnay, 78200 Mantes La Jolie, France; abdelhamid.mekranter@ght-yvelinesnord.fr; 4 Digestive Surgery, Cabinet ADN, 75017 Paris, France; goyerperrine@gmail.com (P.G.); dr.asaget@gmail.com (A.S.); docteur.oberlin@gmail.com (O.O.); 5 Department of Digestive Surgery, Hôpital Franco-Britannique—Fondation Cognacq-Jay, 92300 Levallois-Perret, France; hubert.richa@gmail.com; 6 Department of Pathology and Molecular Biology, IHP Group—Paris, 92240 Malakoff, France; ld@ihp-group.fr; 7 Department of Genetic, Centre Hospitalier Universitaire de Montpellier, 34090 Montpellier, France; p-pujol@chu-montpellier.fr; 8 Department of Radiotherapy, Hartmann Oncology Radiotherapy Group, Cancérologie Paris Ouest, 92300 Levallois-Perret, France; alain.toledano@gmail.com; 9 Department of Gastroenterology, Hôpital La Pitié-Salpétrière, 75013 Paris, France; jean-baptiste.bachet@aphp.fr

**Keywords:** colorectal cancer, circulating tumor DNA, variant allele frequency, *RAS* mutation, comprehensive genomic profiling, liquid biopsy, biomarkers, prognosis, precision medicine, survival analysis

## Abstract

Circulating tumor DNA (ctDNA) analysis offers a non-invasive approach to molecular profiling. While *RAS* mutations are well-established predictive biomarkers in metastatic colorectal cancer (mCRC), the prognostic value of their variant allele frequency (VAF) remains unclear. We retrospectively analyzed individual patient data with mCRC who underwent ctDNA testing using the FoundationOne^®^ Liquid CDx assay. The primary objective was to determine the optimal *RAS* VAF cutoff for overall survival (OS) prognostication. Between November 2020 and July 2024, 282 patients were enrolled. Among 265 eligible patients, 134 (50.6%) were ct*RAS* mutant, 25 (9.4%) ct*BRAF* ^V600E^ mutant, and 106 (40.0%) were ct*RAS/BRAF* wild-type. A *RAS* VAF threshold of 5% yielded the highest prognostic discrimination for OS (HR = 2.41; 95% CI 1.65–3.55; *p* < 0.0001; C-index = 0.601). ct*RAS*-high mutant tumors (VAF ≥ 5%) were associated with synchronous metastatic disease, multiple metastatic sites, higher blood tumor mutational burden, and elevated tumor fraction. ct*RAS*-low mutant tumors (VAF < 5%) were more frequently metachronous, presented with a single metastatic site, and showed liver involvement. High *RAS* VAF in ctDNA is a strong and independent prognostic marker for OS in mCRC. Quantitative ctDNA profiling may enhance risk stratification and guide personalized management strategies.

## 1. Introduction

Colorectal cancer (CRC) is the third most common cancer worldwide and the second leading cause of cancer death, with about 1.9 million new cases and more than 900,000 deaths annually, reflecting a substantial and growing population health burden [1,2]. Global burden analyses based on the Global Burden of Disease framework show sustained increases in CRC disability-adjusted life years from 1990 to 2021, with projections indicating further growth by 2040, underscoring rising morbidity and premature mortality across regions [3]. Advances in molecular profiling have significantly improved our understanding of CRC biology, with mutations in the RAS gene family—particularly V-Ki-ras2 Kirsten rat sarcoma viral oncogene homolog (KRAS) and neuroblastoma RAS viral oncogene homolog (NRAS)—emerging as key biomarkers for guiding therapeutic decisions in metastatic CRC (mCRC) [4,5]. These mutations are known to predict resistance to anti-epithelial growth factor receptor (EGFR) monoclonal antibodies such as cetuximab and panitumumab, and are routinely assessed in clinical practice [6,7,8,9].

Beyond their predictive role, the prognostic significance of *RAS* mutations—especially their variant allele frequency (VAF)—remains insufficiently characterized. Circulating tumor DNA (ctDNA) analysis via liquid biopsy has enabled non-invasive, real-time monitoring of tumor molecular dynamics, offering a more comprehensive view of tumor burden and clonal heterogeneity than traditional tissue biopsies [10,11]. VAF, defined as the proportion of sequencing reads harboring a specific mutation, may reflect tumor burden and heterogeneity.

In this study, we explore the prognostic value of circulating *RAS* VAF in patients with mCRC, focusing on its association with overall survival (OS). By integrating molecular quantification with clinical outcomes, we aim to determine whether *RAS* VAF can serve as a biomarker of prognostic relevance and contribute to improved risk stratification in mCRC.

## 2. Results

### 2.1. Patient Population

**Figure 1:**
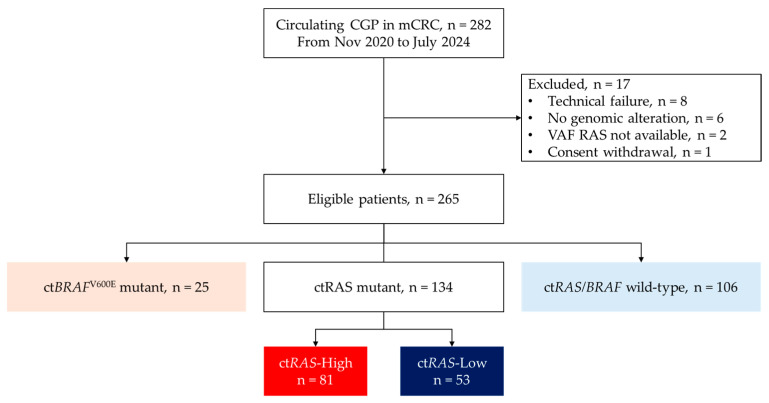
Study Flowchart of Patient Inclusion and Exclusion. Out of 282 patients who underwent ctDNA testing, 17 were excluded due to technical failure (*n* = 8), absence of reportable genomic alterations (*n* = 6), unavailable *RAS* VAF (*n* = 2), or withdrawal consent (*n* = 1). The final cohort included 265 patients.

Between November 2020 and July 2024, liquid biopsy was performed in 282 patients with mCRC. Seventeen patients were excluded due to technical failure (*n* = 8), absence of reportable genomic alterations (*n* = 6), unavailable VAF for *RAS* genes (*n* = 2), or consent withdrawal (*n* = 1). Among 265 eligible patients, 106 patients (40.0%) were ct*RAS/BRAF* wild-type, 134 (50.6%) harbored ct*RAS* mutations, and 25 (9.4%) carried the ct*BRAF*^V600E^ mutation (Figure 1).

### 2.2. Sampling and Blood Analysis

Liquid biopsies were performed in first-line setting in 149 (56.2%) patients and second- or subsequent line setting in 116 (43.8%) patients. The median turnaround time was 12.0 days (range 7.0 to 23.0).

### 2.3. Determination of VAF Threshold

**Figure 2:**
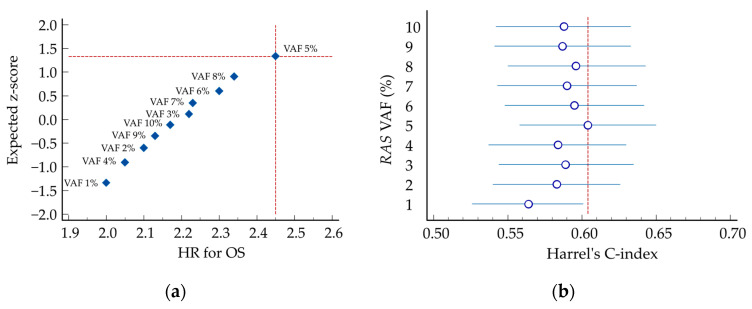
Distribution of *RAS* Variant Allele Frequency (VAF) in ctDNA. (**a**) Normal plot showing the distribution of *RAS* VAF across the 134 ct*RAS*-mutant patients. The horizontal axis of the Normal plot shows the hazard ratio (HR) for death, and the vertical axis shows the corresponding expected number of standard deviations from the mean (z-score), based on the ranks of the observed values; (**b**) Forest plot. The horizontal axis shows the observed values of Harrel’s C-index, and the vertical axis shows the value of variant allele frequency (VAF, %) of *RAS* genes. The central values are represented by markers and the confidence intervals by horizontal lines.

The cut-off corresponding to VAF 5% yielded the most significant separation in OS (*p* < 0.0001), with a HR for death in the high-expression group of 2.41 (95% CI 1.63 to 3.55), indicating an unfavorable prognostic association. Moreover, this cut-off also yielded the highest concordance index (C-index 0.601, 95% CI 0.555–0.648) (Table 1, Figure 2).

**Figure 3:**
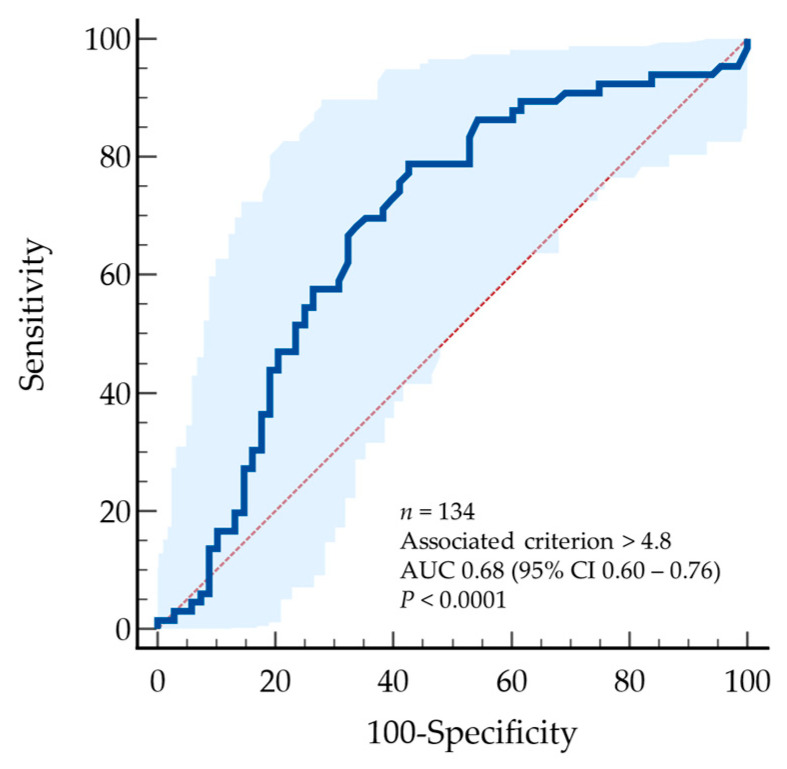
Receiver operating characteristic (ROC) analysis for 1-year survival (1-yr OS).

To strengthen the definition of the 5% VAF threshold, we performed a receiver operating characteristic (ROC) analysis for OS. The ROC curve yielded an area under the curve (AUC) of 0.681 (95% Boodstrap CI 0.590 to 0.769; *p* < 0.0001), indicating moderate discriminatory ability. The Youden index identified an optimal cutoff at >4.8% (95% CI > 2.15 to >16.2) with sensitivity and specificity rate s of 78.8 and 57.4 (Figure 3).

ROC curve presents a plot of the true positive rate (Sensitivity) in function of the false positive rate (100-Specificity) for different cut-off points of *RAS* VAF. Each point on the ROC curve represents a sensitivity/specificity pair corresponding to a particular decision threshold.

Based on these findings, the 5% value of VAF for *RAS* genes was selected as the optimal cut-off for subsequent analyses. The mean VAFs were 1.5% (SD 1.4) and 25.9% (SD 18.8) in patients with ct*RAS*-low and ct*RAS*-high tumors, respectively.

### 2.4. Patient and Tumor Characteristics

Patient and tumor characteristics at the time of circulating CGP are summarized in Table 2. The study population included both sexes, with a nearly balanced distribution: 49% female and 51% male patients. The overall mean age of patients was 64.4 years (SD 13.5), while ct*RAS* mutant patients had a mean age of 65.0 years (SD 12.7).

The ct*RAS*-low (VAF < 5%) and ct*RAS*-high (VAF ≥ 5%) subgroups exhibited significant clinical and biological divergence. ct*RAS*-low tumors were more commonly associated with metachronous metastatic presentation (*p* = 0.046), a solitary metastatic site (*p* = 0.017), and predominant hepatic involvement (*p* = 0.001). Conversely, ct*RAS*-high tumors exhibited significantly elevated blood tumor mutational burden (bTMB; *p* < 0.0001) and increased TF (*p* < 0.0001). Metachronous dissemination was more frequently observed in ct*RAS*-low and ct*RAS/BRAF* wild-type profiles, whereas synchronous metastatic disease was significantly enriched in ct*RAS*-high and ct*BRAF*-mutant cases (*p* = 0.020).

Patients with ct*RAS*-mutant tumors exhibited distinct clinical and molecular characteristics across treatment lines, with high-grade histology, prior resection of the primary tumor, presence of multiple metastatic sites, pulmonary involvement, and elevated bTMB occurring more frequently in those receiving second-line or subsequent therapies compared to those treated in the first-line setting (Table A1).

### 2.5. Prognostic Analysis of VAF

**Figure 4:**
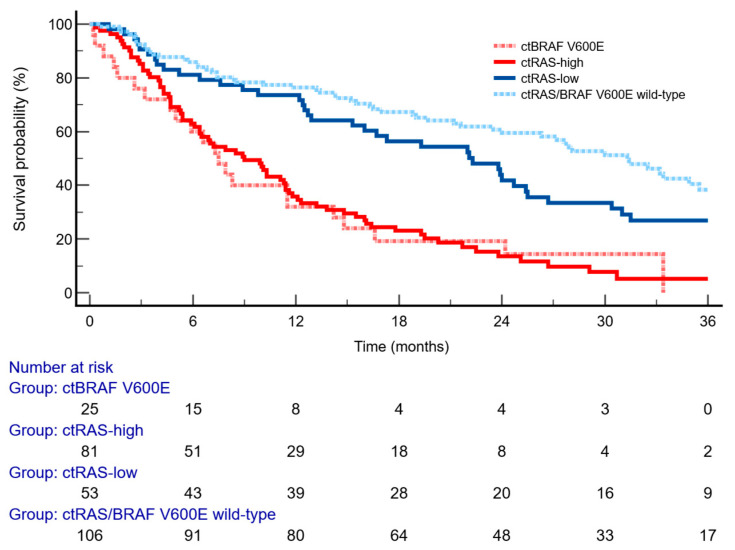
Kaplan–Meier curves for overall survival stratified by molecular subgroups and VAF *RAS* level (light blue dotted line, RAS/BRAF wild-type; dark blue solid line, RAS mutant VAF-Low; red solid line, RAS mutant VAF-High; pink dotted line, BRAF^V600E^ mutant). The high-expression group demonstrated significantly poorer survival compared to the low-expression group (log-rank *p* < 0.0001; HR = 2.41; 95% CI 1.63 to 3.55).

The median follow-up for OS was 35.3 months (95% CI 32.1–58.6). Stratification using a *RAS* VAF threshold of 5% revealed a significantly increased risk of death, with a HR of 2.41 (95% CI 1.63–3.55; *p* < 0.0001) (Table 1, Figure 4). No statistically significant difference in OS was observed between the ct*RAS/BRAF* wild-type and ct*RAS*-low subgroups (HR = 1.40; 95% CI 0.98–2.00). Moreover, patients with ct*RAS*-high mutant tumors exhibited survival outcomes comparable to those with ct*BRAF*^V600E^ mutations (HR = 0.98; 95% CI 0.51–1.91).

**Figure 5:**
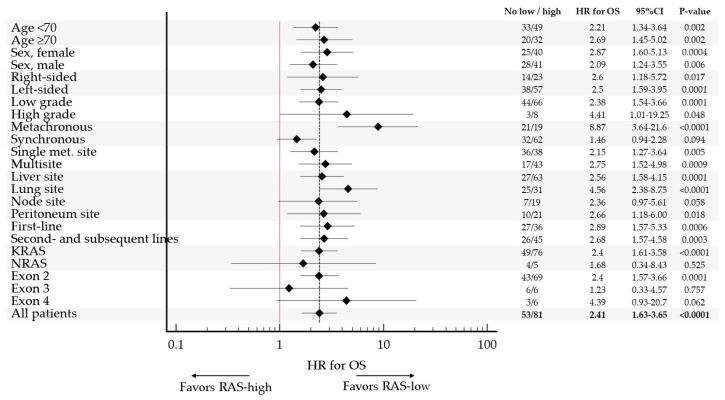
Forest Plot of Sensitivity Analyses. Forest plot showing hazard ratios for overall survival associated with high *RAS* VAF (≥5%) across predefined subgroups (e.g., age, sex, metastatic sites, treatment line), confirming consistent prognostic impact.

The poor prognostic impact of *RAS*-high on OS was observed in all pre-specified subgroups, confirming consistent prognostic impact of VAF *RAS* in mCRC (Table A2, Figure 5).

The strongest prognostic value of VAF *RAS* was observed in patients with metachronous metastatic disease (HR 8.87, 95% CI 3.64 to 21.6), lung involvement (HR 4.56, 95% CI 2.38 to 8.75) and high-grade tumors (HR 4.41, 95% CI 1.01 to 19.25). Of note, there was no statistically significant association between VAF *RAS* and OS in a small subset of patients with either *NRAS* (*n* = 9) or exon 3 (*n*= 12) tumors.

In first-line setting, median OS were 31.5 months (95% CI 12.5 to 31.5) and 11.6 months (95% CI 6.4 to 19.3) in ct*RAS*-low and ct*RAS*-high, respectively (HR 2.89, 95% CI 1.57 to 5.33; *p* = 0.0006). In later lines, median OS were 16.7 months (95% CI 9.8 to 31.0) and 7.2 months (95% CI 4.7 to 23.8) in ct*RAS*-low and ct*RAS*-high, respectively (HR 2.68, 95% CI 1.57 to 4.58; *p* = 0.0003) (Table A1).

Multivariate analysis was conducted incorporating variables that demonstrated statistical significance in univariate assessments (Table A2). Independent adverse prognostic factors for OS included elevated (≥10%) tumor fraction (HR 2.58, 95% CI 1.69–3.95; *p* < 0.0001), administration of second- or later treatment line (HR 2.46, 95% CI 1.61–3.75; *p* < 0.0001), and increased (≥10) bTMB (HR 1.71, 95% CI 1.09–2.70; *p* = 0.021).

### 2.6. Molecular Characteristics and Co-Occuring Alterations

ct*RAS*-high mutant tumors were associated with higher bTMB (*p* < 0.0001) and higher TF (*p* < 0.0001) than ct*RAS*-low mutant tumors. There was no difference between the 2 subgroups in terms of location or type of mutation. Both ct*APC* and ct*TP53* mutant high (VAF ≥ 5%) tumors were significantly more frequent in patients with ct*RAS*-high than ct*RAS*-low tumors. Significant differences were observed in terms of molecular co-alterations between the 2 groups (*p* = 0.006), TGFβ and Wnt/β-catenin pathways being more frequently altered in patients with ct*RAS*-high than ct*RAS*-low tumors (Table 3).

## 3. Discussion

This study demonstrates that the VAF of *RAS* mutations detected via ctDNA is a robust prognostic biomarker for OS in patients with mCRC. Stratifying patients based on the value of 5% VAF for *RAS* genes provided the most significant prognostic discrimination for OS. This threshold yielded a clear separation between survival curves, with a HR for death of 2.41 indicative of a clinically meaningful increase in mortality risk among patients with higher VAF value (≥5%).

Although the cut-off was selected through exploratory testing rather than a statistically corrected optimization procedure, the approach remains valid within a clinical research framework, particularly when supported by biological plausibility and reproducibility across subgroups. The prognostic significance of *RAS* VAF was particularly consistent across treatment settings, with ctRAS-high status associated with significantly reduced OS both in first-line (HR 2.89) and later-line therapies (HR 2.68), indicating that its adverse prognostic effect is preserved irrespective of treatment line. Furthermore, in the context of metachronous metastatic disease, a high *RAS* VAF was associated with particularly poor prognosis, reflected by a markedly elevated HR for death of 8.87. These findings underscore the clinical relevance of quantitative ctDNA metrics beyond mere *RAS* mutation status. For clinical translation, we selected 5% as the operational threshold, which closely aligns with the statistically derived value while offering a pragmatic and reproducible benchmark across laboratories. While the discriminatory power is modest, the validated threshold provides a clinically feasible tool for risk stratification and highlights the potential of quantitative ctDNA metrics to complement existing prognostic markers in mCRC.

High *RAS* VAF tumors were associated synchronous presentation, multiple metastatic sites, and elevated tumor burden—as evidenced by higher bTMB and TF. Additionally, ct*RAS*-high mutant tumors were associated with molecular co-alterations from TGFβ or Wnt/β-catenin pathways. Conversely, ct*RAS*-low tumors were more frequently metachronous, limited to a single metastatic site, and predominantly involved the liver. Our findings should be interpreted as demonstrating a statistical association between higher ctDNA-based *RAS* VAF and poorer survival outcomes, rather than implying a biological causal role of *RAS* mutations in driving tumor aggressiveness. While *RAS* mutations are well-established oncogenic events in colorectal cancer, the quantitative burden detected in plasma likely reflects tumor load and shedding dynamics rather than intrinsic biological behavior. Thus, VAF ≥ 5% should be considered a prognostic biomarker that correlates with disease state, not a mechanistic determinant of progression. Future studies integrating functional assays and longitudinal sampling will be required to explore whether quantitative mutation burden has any direct biological implications.

The use of liquid biopsy offers several advantages, including non-invasive sampling, real-time monitoring, and the ability to capture tumor heterogeneity [12,13,14]. Importantly, our sensitivity analyses confirmed the prognostic value of high *RAS* VAF across all clinical subgroups, reinforcing its potential utility in routine risk stratification. These findings may inform clinical decision-making, particularly in considering early therapeutic escalation for patients with high ct*RAS* VAF.

In previous studies, it has been shown that ctDNA metrics provide deeper insights into tumor biology, reflecting disease aggressiveness and burden [15]. Moreover, ctDNA metrics are prognostic for survival. In the RASANC study, ctDNA mutant allele frequency—defined as the highest observed frequency among mutated genes—demonstrated significant prognostic relevance [16]. At the optimal cut-off of 20%, the HR for death was 2.58 (95% CI 2.02 to 3.28). In a translational analysis of the Valentino study, the maximum VAF detected among mutated genes was taken as an estimate of the ctDNA amount in patients with *RAS* wild-type mCRC [17]. Patients with high VAF (superior to the median of 12.6%) presented with liver metastases and synchronous presentation, and had poorer survival compared to those with low VAF (HR: 1.82, 95% CI 1.20 to 2.76). Together, these studies underscore the potential of integrating quantitative ctDNA analyses into routine practice, both as a baseline prognostic tool and as a dynamic biomarker to guide therapeutic strategies.

*RAS* mutations are a common oncogenic driver, but their frequency, isoform distribution, and clinical impact vary considerably across different tumor types. In CRC, both *KRAS* and *NRAS* mutations occur (*KRAS* ~40%, *NRAS* ~3–5%), with diverse codon hotspots (G12, G13, Q61). In pancreatic ductal adenocarcinoma, *KRAS* mutations dominate (~90–95%), especially at codon 12 (G12D, G12V, G12R), while *NRAS* and *HRAS* mutations are essentially absent [18]. *RAS* mutations are rare in renal cell carcinoma (RCC) [19]. When present, they are usually confined to specific histologic subtypes such as papillary renal neoplasms with reverse polarity, where *KRAS* mutations occur at high frequency. In the broader RCC spectrum, *KRAS* mutations affect only about 1% of cases, while *NRAS* and *HRAS* mutations are essentially absent. Thus, unlike colorectal or pancreatic cancers, *RAS* mutations are not a major driver in renal cancer, but when detected, they may define distinct molecularly characterized subgroups.

Limitations of this study include its retrospective design, treatment heterogeneity, and the absence of longitudinal ctDNA testing. First, we acknowledge the limitations inherent to retrospective designs, which inherently restrict the strength of causal inferences and may introduce selection bias. To mitigate selection bias, we included all consecutive patients with mCRC who underwent ctDNA testing at our center during the study period. Exclusion criteria were strictly technical or ethical (e.g., consent withdrawal), and no patients were excluded based on clinical outcomes or treatment response. Second, patient heterogeneity, including differences in prior treatments and disease characteristics, could have influenced survival outcomes and confounded the observed associations. The cohort analyzed in this study was derived from a single-center patient population, which may not fully reflect the heterogeneity of broader mCRC populations. While treatment heterogeneity exists, our primary endpoint—OS—was chosen to minimize confounding from specific therapeutic regimens. Sensitivity analyses across subgroups confirmed the prognostic impact of *RAS* VAF regardless of treatment line. Additionally, while the FoundationOne^®^ Liquid CDx assay offers standardized quantification, inter-platform variability in VAF measurement warrants further validation across different technologies. VAF values were analyzed as reported by the assay without adjustment for estimated tumor fraction. As tumor fraction correlates with ctDNA burden, non-corrected values may overestimate mutation frequency. This limitation highlights the need for future studies to integrate tumor fraction estimates to refine the prognostic utility of *RAS* VAF. Finally, the absence of an independent validation cohort limits the generalizability of our findings, underscoring the need for prospective, multicenter studies to confirm the prognostic utility of *RAS* VAF in ctDNA. Future research should therefore focus on standardized inclusion criteria and independent validation to strengthen the clinical applicability of these results. Beyond ctDNA profiling, nanoscale technologies such as nanomaterials and biosensors are being developed to enhance CRC monitoring, offering complementary sensitivity and theranostic potential [20,21].

These findings suggest that circulating *RAS* VAF may serve as a useful stratification tool in future trials and could inform risk-adapted treatment strategies. Further validation in independent cohorts and prospective studies is warranted to confirm its prognostic utility and generalizability.

## 4. Materials and Methods

### 4.1. Study Design and Patient Population

This retrospective study included adult (≥18 years old) patients diagnosed with mCRC, irrespective of treatment line, who underwent liquid biopsy for ctDNA testing as part of routine molecular profiling. All patients provided written informed consent prior to blood collection, in accordance with ethical standards and institutional guidelines. Patients were treated according to standard-of-care protocols. Clinical data were collected from electronic medical records (DxCare software, version 8.2021.2.8, Dedalus, Antony, France).

### 4.2. Circulating Comprehensive Genomic Profiling (CGP)

Blood sampling was centralized at Hôpital Franco-Britannique—Fondation Cognacq-Jay (Levallois-Perret, France) and was done using FoundationOne^®^ Liquid CDx cfDNA blood collection tubes (8.5 mL nominal fill volume per tube, 2 tubes per patient). The FoundationOne^®^ Liquid CDx assay was performed using cfDNA isolated from plasma derived from anti-coagulated peripheral whole blood [22]. Extracted cfDNA undergoes whole-genome shotgun library construction and hybridization-based capture of 324 cancer-related genes including coding exons and select introns of 309 genes, as well as only select intronic regions or non-coding regions of 15 genes. VAFs, representing the fraction of sequencing reads in which a variant is observed, were reported as measured by the assay and were not adjusted for estimated tumor fraction. In cases where multiple RAS mutations were detected in a single patient, the mutation with the highest VAF was selected for analysis. The ctDNA fraction (TF), the blood tumor mutational burden (bTMB) and microsatellite instability (MSI) were also evaluated (Appendix A.1) [22,23].

### 4.3. Molecular Co-Alterations and Pathways

Selected genes were classified into seven prespecified molecular pathways: mitogen activated protein kinase (MAPK), phosphoinositide 3 kinase/protein kinase B/mammalian target of rapamycin (PIK3/AKT/mTOR), DNA damage repair (DDR) system, transforming growth factor beta (TGFβ), Wingless-related integrated site (Wnt), cell cycle, and immune (Appendix A.2). Regarding genomic co-alterations, mutation status was dichotomized as mutated versus non-mutated, irrespective of the VAF level. When multiple co-alterations were present, the variant with the highest VAF was retained.

### 4.4. Statistical Analysis

To evaluate the prognostic impact of the VAF of *RAS* genes on survival, we performed a series of manual dichotomizations based on VAF amount. For each dichotomization, patients were stratified into low and high groups, and survival outcomes were compared using Kaplan–Meier estimates and the log-rank test. Harrell’s C-index were also calculated [24]. The confidence interval (CI) of Harrell’s C-index was calculated using the modified τ method [25]. The cut-off yielding the most significant separation between survival curves—defined by a clinically meaningful hazard ratio (HR), the lowest log-rank *p*-value and the highest C-index—was selected as the optimal threshold. This approach, although exploratory, allowed for transparent assessment of the variable’s discriminative capacity while maintaining interpretability within a clinical context. ROC curve analysis with bootstrap resampling was performed to confirm the optimal threshold for *RAS* VAF in relation to OS.

Demographic, clinical, and genomic characteristics were obtained using descriptive statistics. Categorical variables were summarized in numbers and percentages, continuous variables were presented as means with standard deviation (SD) and medians with minima and maxima. OS was defined as the time interval between the date of liquid biopsy and the date of death from any cause. Alive patients were censored to the last date known to be alive. Kaplan–Meier method was used for survival, the reverse Kaplan–Meier method for follow-up, and Cox regression for multivariate analyses. A *p* value < 0.05 was considered statistically significant. All analyses were conducted using MedCalc^®^ Statistical Software version 22.021 (MedCalc Software Ltd., Ostend, Belgium; https://www.medcalc.org; 2023).

## 5. Conclusions

In conclusion, our study highlights the prognostic significance of *RAS* VAF in ctDNA, with a ≥5% threshold identifying patients at higher risk of poor survival. Integrating quantitative ctDNA metrics into clinical practice may enhance personalized management strategies in mCRC. Future research should aim to validate the prognostic utility of *RAS* VAF thresholds in larger, prospective multicenter cohorts to strengthen their clinical applicability.

## Figures and Tables

**Table 1:** Determination of VAF *RAS* cut-off.

VAF Cut-Off (%)	No. Low	No. High	HR	HR 95% CI	*p*-Value	C-Index	C-Index 95% CI
1	25	109	1.99	1.29–3.06	0.0018	0.560	0.522–0.597
2	35	99	2.07	1.40–3.08	0.0003	0.580	0.537–0.623
3	43	91	2.19	1.48–3.22	0.0001	0.586	0.541–0.632
4	49	85	2.02	1.37–2.96	0.0003	0.581	0.534–0.628
5	53	81	2.41	1.63–3.55	<0.0001	0.601	0.555–0.648
6	61	73	2.26	1.53–3.34	<0.0001	0.593	0.545–0.640
7	62	72	2.19	1.48–3.24	0.0001	0.588	0.540–0.636
8	66	68	2.29	1.54–3.42	<0.0001	0.594	0.547–0.641
9	71	63	2.09	1.40–3.13	0.0003	0.585	0.538–0.631
10	74	60	2.12	1.41–3.19	0.0003	0.586	0.540–0.632

**Table 2:** Patient and tumor characteristics.

Covariate	All Patients	ct*BRAF*	ct*RAS*-High (VAF ≥ 5%)	ct*RAS*-Low (VAF < 5%)	ct*RAS/BRAF* WT	*p*-Value (All)	*p*-Value (*RAS*)
No of patients	265	25	81	53	106		
Age, years						0.099	0.838
<70	163 (61.5)	10 (40.0)	49 (60.5)	33 (62.3)	71 (67.0)		
≥70	102 (38.5)	15 (60.0)	32 (39.5)	20 (37.7)	35 (33.0)		
Sex						0.699	0.803
Female	130 (49.1)	15 (60.0)	40 (49.4)	25 (47.2)	50 (47.2)		
Male	135 (50.9)	10 (40.0)	41 (50.6)	28 (52.8)	56 (52.8)		
Tumor sidedness						<0.0001	0.930
Right-sided	85 (32.1)	20 (80.0)	23 (28.4)	14 (26.4)	28 (26.4)		
Left-sided	175 (66.0)	5 (20.0)	57 (70.4)	38 (71.7)	75 (70.8)		
Both	5 (1.9)	0	1 (1.2)	1 (1.9)	3 (2.8)		
Grading						0.016	0.411
Low	207 (87.7)	14 (66.7)	66 (89.2)	44 (93.6)	83 (88.3)		
High	29 (12.3)	7 (33.3)	8 (10.8)	3 (6.4)	11 (11.7)		
Missing	29	4	7	6	12		
Initial stage						0.020	0.046
Non metastatic	86 (32.5)	4 (16.0)	19 (23.5)	21 (39.6)	42 (39.6)		
Metastatic	179 (67.5)	21 (84.0)	62 (76.5)	32 (60.4)	64 (60.4)		
Primary resection						0.089	0.028
No	62 (23.4)	7 (28.0)	26 (32.1)	8 (15.1)	21 (19.8)		
Yes	203 (76.6)	18 (72.0)	55 (67.9)	45 (84.9)	85 (80.2)		
Disease stage						0.107	-
Locally advanced	4 (1.5)	0	0	0	4 (3.8)		
Metastatic	261 (98.5)	25 (100.0)	81 (100.0)	53 (100.0)	102 (96.2)		
No of met. Sites						0.002	0.017
0–1	151 (57.0)	8 (32.0)	38 (46.9)	36 (67.9)	69 (65.1)		
>1	114 (43.0)	17 (68.0)	43 (53.1)	17 (32.1)	37 (34.9)		
Liver involvement						0.009	0.001
No	90 (34.0)	11 (44.0)	18 (22.2)	26 (49.1)	35 (33.0)		
Yes	175 (66.0)	14 (56.0)	63 (77.8)	27 (50.9)	71 (67.0)		
Lung involvement						0.004	0.309
No	179 (67.5)	17 (68.0)	50 (61.7)	28 (52.8)	84 (79.2)		
Yes	86 (32.5)	8 (32.0)	31 (38.3)	25 (47.2)	22 (20.8)		
Node involvement						0.029	0.369
No	207 (78.1)	14 (56.0)	62 (76.5)	44 (83.0)	87 (82.1)		
Yes	58 (21.9)	11 (44.0)	19 (23.5)	9 (17.0)	19 (17.9)		
Peritoneal involvement						0.124	0.345
No	197 (74.3)	14 (56.0)	60 (74.1)	43 (81.1)	80 (75.5)		
Yes	68 (25.7)	11 (44.0)	21 (25.9)	10 (18.9)	26 (24.5)		
Treatment setting						0.019	0.463
First-line	149 (56.2)	16 (64.0)	36 (44.4)	27 (50.9)	70 (66.0)		
Subsequent lines	116 (43.8)	9 (36.0)	45 (55.6)	26 (49.1)	36 (34.0)		

**Table 3:** Molecular characteristics of ct*RAS* mutant tumors according to VAF level (ct*RAS*-low, VAF < 5%; ct*RAS*-high, VAF ≥ 5%).

Covariate	All *RAS*	ct*RAS*-High	ct*RAS*-Low	*p*-Value
No of patients	134	81	53	
ctMSI				0.251
Detected	2 (1.5)	2 (2.5)	0	
Not detected	132 (98.5)	79 (97.5)	53 (100.0)	
Blood TMB, Mut/Mb				<0.0001
<5	54 (40.3)	19 (23.5)	35 (66.0)	
5 to 10	48 (35.8)	35 (43.2)	13 (24.5)	
≥10	32 (23.9)	27 (33.3)	5 (9.4)	
Tumor fraction, %				<0.0001
<10	58 (43.3)	11 (13.6)	47 (88.7)	
≥10	76 (56.7)	70 (86.4)	6 (11.3)	
Codon				0.335
12	88 (65.7)	51 (63.0)	37 (69.8)	
13	24 (17.9)	18 (22.2)	6 (11.3)	
22	1 (0.7)	0	1 (1.9)	
61	11 (8.2)	5 (6.2)	6 (11.3)	
117	1 (0.7)	1 (1.2)	0	
146	9 (6.7)	6 (7.4)	3 (5.7)	
Selected *RAS* variants				
G12A	12 (9.0)	3 (3.7)	9 (17.0)	0.009
G12C	8 (6.0)	4 (4.9)	4 (7.5)	0.535
G12D	34 (25.4)	20 (24.7)	14 (26.4)	0.823
G12V	26 (19.4)	18 (22.2)	8 (15.1)	0.309
*APC*				<0.0001
Wild-type	16 (11.9)	8 (9.9)	8 (15.1)	
Mutant-Low	43 (32.1)	7 (8.6)	36 (67.9)	
Mutant-High	75 (56.0)	66 (81.5)	9 (17.0)	
*TP53*				<0.0001
Wild-type	22 (16.4)	7 (8.6)	15 (28.3)	
Mutant-Low	38 (28.4)	8 (9.9)	30 (56.6)	
Mutant-High	74 (55.2)	66 (81.5)	8 (15.1)	
Co-altered pathways				0.006
MAPK	11 (8.2)	6 (7.4)	5 (9.4)	
PIK3/AKT/mTOR	28 (20.9)	19 (23.5)	9 (17.0)	
TGFβ	19 (14.2)	16 (19.8)	3 (5.7)	
Wnt/β-catenin	10 (7.5)	10 (12.3)	0	
DNA damage repair	9 (6.7)	6 (7.4)	3 (5.7)	
Cell cycle progression	4 (3.0)	2 (2.5)	2 (3.8)	
Immune	3 (2.2)	2 (2.5)	1 (1.9)	
Other	24 (17.9)	11 (13.6)	13 (24.5)	
None	26 (19.4)	9 (11.1)	17 (32.1)	

## Data Availability

The data supporting the findings of this study are available on request from the corresponding author. The data are not publicly available due to their containing information that could compromise the privacy of research participants.

## Abbreviations

The following abbreviations are used in this manuscript:

CRC	Colorectal cancer
*KRAS*	V-Ki-ras2 Kirsten rat sarcoma viral oncogene homolog
*NRAS*	neuroblastoma RAS viral oncogene homolog
mCRC	Metastatic colorectal cancer
*EGFR*	Epithelial Growth Factor Receptor
VAF	Variant allele frequency
cfDNA	Circulating free DNA
ctDNA	Circulating tumor DNA
OS	Overall survival
ROC	Receiver operating characteristic
AUC	Area under curve
bTMB	Blood tumor mutational burden
TF	Tumor fraction
MSI	Microsatellite instability
CI	Confidence interval
HR	Hazard ratio
SD	Standard deviation
CGP	Comprehensive genomic profiling
*APC*	Adenomatous polyposis coli
*TP53*	Tumor protein 53
*MAPK*	Mitogen activated protein kinase
*PIK3*	Phosphoinositide 3 kinase
*AKT*	Protein kinase B
mTOR	Mammalian target of rapamycin
DDR	DNA damage repair
*TGFβ*	Transforming growth factor beta
Wnt	Wingless-related integrated site
RCC	Renal cell carcinoma

## Appendix A

### Appendix A.1. Description of Genomic Signatures Determination

ctDNA fraction (TF)

TF is a determination of the amount of ctDNA as a fraction of total cfDNA in a blood sample. ctDNA TF was quantified by combining multiple methods. For samples with significant aneuploidy, the purity assessment from a robust copy-number model that accounts for both the observed coverage variation and allele frequencies of genome-wide SNP allele frequencies is used to determine the TF estimate. When significant aneuploidy is not present, the allele frequencies of short variants and rearrangements deemed very likely to be somatic are used to estimate TF. In addition, an assessment of cfDNA fragment sizes was used to exclude clonal hematopoiesis (CH)-derived aneuploidy from copy-number modeling.

Blood Tumor Mutational Burden (bTMB)

bTMB is measured by counting all synonymous and non-synonymous variants present at 0.5% allele frequency or greater and filtering out potential germline variants according to published databases of known germline polymorphisms including dbSNP and ExAC. Additional germline alterations are assessed for potential germ line status and filtered out using a somatic-germline/zygosity algorithm. Furthermore, known and likely driver mutations are filtered out to exclude bias of the data set. The resulting mutation number is then divided by the coding region corresponding to the number of total variants counted, or approximately 750 kilobases (kb). The resulting number is reported in units of mutations per megabase (mut/Mb).

Microsatellite instability (MSI) signature

To determine MSI status, approximately 2000 repetitive loci (minimum of 5 repeat units of mono-, di-, and trinucleotides) were assessed to determine what repeat lengths are present in the sample. A locus containing a repeat length present in an internal database generated using >3000 clinical samples was considered to be ’unstable’. An MSI indicator is generated by calculating the fraction of unstable loci, considering only those loci that achieve adequate coverage for consideration for the sample. Samples with >0.5% unstable loci were considered to be MSI-High.

### Appendix A.2. List of Genes or Genomic Signatures of Seven Prespecified Genomic Pathways

**Pathways**	**Genes**
MAPK	*ARAF*, *RAF1*, *ERBB1 (EGFR)*, *ERBB2 (HER2)*, *ERBB3 (HER3)*, *ERBB4*, *FLT3*, *FGFR1-4*, *HRAS*, *HGF*, *MAP2K1 (MEK1)*, *MAP2K2 (MEK2)*, *MAP2K4*, *MAP3K1*, *MAP3K13*, *MAPK1*, *MET*, *MST1R*, *NF1*
PIK3/AKT/mTOR	*AKT1-3*, *ARID1A*, *CCND2*, *CTNNB1*, *FBXW7*, *FLCN*, *MTOR*, *PIK3CA*, *PRKAR1A*, *PTEN*, *RPTOR*, *STK11*, *TSC2*
DDR	*ATM*, *BAP1*, *BARD1*, *BRCA1-2*, *BRIP1*, *CHEK2*, *FANCA*, *FANC*, *FANCG*, *FANCL*, *INPP4B*, *MRE11A*, *MUTYH*, *NBN*, *PALB2*, *RAD51*, *RAD51B*, *RAD51C*, *RAD51D*, *SETD2*, *STAG2*
TGFβ	*KDM5C*, *SMAD2*, *SMAD4*, *TGFBR2*
Wnt	*FAM123B*, *CDC73*, *CDK8*, *CTNNB1*, *MYC*, *RNF43*
Cell cycle	*CCND3*, *CCNE1*, *CDK4*, *CDK6*, *CDKN1B*, *CDKN2AB*, *GATA3*, *KDM5A*, *MEN1*, *PARK2*, *PPPR1A*, *QKI*, *RB1*, *SMARCA4*, *SMARCB1*
Immune	ctMSI, bTMB-high (≥20), *CD274* (*PD-L1*), *MLH1*, *MSH2*, *MSH3*, *MSH6*, *PMS2*, *POLD1*, *POLE*

## Appendix B

**Table A1:** Clinical and molecular characteristics in ct*RAS* mutant group according to treatment setting.

Covariate	All Patients	First-Line	Second- and Later Lines	*p*-Value
No of patients	134	63	71	
Age, years				0.209
<70	82 (61.2)	35 (55.6)	47 (66.2)	
≥70	52 (38.8)	28 (44.4)	24 (33.8)	
Sex				0.219
Female	65 (48.5)	27 (42.9)	38 (53.5)	
Male	69 (51.5)	36 (57.1)	33 (46.5)	
Tumor sidedness				0.371
Right-sided	37 (27.6)	21 (33.3)	16 (22.5)	
Left-sided	95 (70.9)	41 (65.1)	54 (76.1)	
Both	2 (1.5)	1 (1.6)	1 (1.4)	
Grading				0.030
Low	110 (82.1)	55 (87.3)	55 (77.5)	
High	11 (8.2)	1 (1.6)	10 (14.1)	
Missing	13 (9.7)	7 (11.1)	6 (8.5)	
Initial stage				0.942
Non metastatic	94 (70.1)	44 (69.8)	50 (70.4)	
Metastatic	40 (29.9)	19 (30.2)	21 (29.6)	
Primary resection				<0.0001
No	34 (25.4)	27 (42.9)	7 (9.9)	
Yes	100 (74.6)	36 (57.1)	64 (90.1)	
No of met. Sites				0.031
0–1	74 (55.2)	41 (65.1)	33 (46.5)	
>1	60 (44.8)	22 (34.9)	38 (53.5)	
Liver involvement				0.324
No	44 (32.8)	18 (28.6)	26 (36.6)	
Yes	90 (67.2)	45 (71.4)	45 (63.4)	
Lung involvement				0.027
No	78 (58.2)	43 (68.3)	35 (49.3)	
Yes	56 (41.8)	20 (31.7)	36 (50/7)	
Node involvement				0.945
No	106 (79.1)	50 (79.4)	56 (78.9)	
Yes	28 (20.9)	13 (20.6)	15 (21.1)	
Peritoneal involvement				0.814
No	103 (76.9)	49 (77.8)	54 (76.1)	
Yes	31 (23.1)	14 (22.2)	17 (23.9)	
bTMB, Mut/Mb				0.004
<10	102 (76.1)	55 (87.3)	47 (66.2)	
≥10	32 (23.9)	8 (12.7)	24 (33.8)	
Tumor fraction, %				0.926
<10	58 (43.3)	27 (42.9)	31 (43.7)	
≥10	76 (56.7)	36 (57.1)	40 (56.3)	
Exon				0.708
2	112 (84.2)	53 (85.3)	59 (83.1)	
3–4	21 (15.8)	9 (14.5)	12 (16.9)	
VAF, %				0.463
<5	53 (39.6)	27 (42.9)	26 (36.6)	
≥5	81 (60.4)	36 (57.1)	45 (63.4)	

**Table A2:** Univariate and multivariate analysis for OS in ct*RAS* mutant subgroup.

Covariate			Univariate			Multivariate		
Covariate	No of Patients	No of Events	HR	95% CI	*p*-Value	HR	95% CI	*p*-Value
Age, years								
<70	82	65						
≥70	52	45	1.40	0.94–2.08	0.099			
Sex								
Female	65	51						
Male	69	59	0.98	0.67–1.42	0.898			
Tumor sidedness								
Right-sided	37	27						
Left-sided	95	81	1.48	0.99–2.21				
Both	2	2	4.99	0.34–73.8	0.026			
Grading								
Low	110	90						
High	11	10	1.33	0.64–2.79				
Missing	13	10	0.95	0.51–1.80	0.666			
Initial stage								
Non metastatic	40	27						
Metastatic	94	83	1.69	1.14–2.51	0.009			
Primary resection								
No	34	30						
Yes	100	80	0.51	0.31–0.84	0.008			
No of met. Sites								
0–1	74	59						
>1	60	51	1.22	0.83–1.79	0.304			
Liver involvement								
No	44	36						
Yes	90	74	1.13	0.76–1.66	0.564			
Lung involvement								
No	78	60						
Yes	56	50	1.26	0.86–1.85	0.237			
Node involvement								
No	106	88						
Yes	28	22	0.85	0.54–1.33	0.474			
Peritoneal involvement								
No	103	84						
Yes	31	26	1.21	0.76–1.93	0.414			
Treatment setting								
First-line	63	44						
Subsequent lines	71	66	2.28	1.54–3.38	<0.0001	2.46	1.61–3.75	<0.0001
bTMB, Mut/Mb								
<10	102	79						
≥10	32	31	4.06	2.30–7.16	<0.0001	1.71	1.09–2.70	0.021
Tumor fraction, %								
<10	58	41						
≥10	76	69	2.59	1.75–3.83	<0.0001	2.58	1.69–3.95	<0.0001
Exon								
2	112	92						
3–4	21	18	1.40	0.80–2.47	0.239			
VAF, %								
<5	53	37						
≥5	81	73	2.41	1.63–3.55	<0.0001			
						C-index 0.68	0.63–0.74	<0.0001
